# Joint Promotion of Online Retail Based on Promotional Effort under the Shopping Festival Background

**DOI:** 10.1155/2022/6744565

**Published:** 2022-09-28

**Authors:** Jinghua Zhao, Wei Zhang, Juan Feng, Yan Zhang, Shujiao Ma

**Affiliations:** ^1^School of Business, University of Shanghai for Science and Technology, Shanghai, China; ^2^College of Business Administration, Zhejiang University of Finance and Economics, Hangzhou, China

## Abstract

With the prevalence of shopping festivals launched by e-commerce platforms, online retailers and customers are gradually showing signs of fatigue. In order to enrich the promotion strategies of the shopping festivals, break the bottleneck, and achieve mutual benefit, this paper proposes three promotional models based on platform-based promotional effort, namely, the joint promotion model with no promotional effort, the joint promotion model with only the platform putting in the promotional effort, and the joint promotion model with the platform and the e-retail sharing the promotional effort. The study shows that the platform and the online retailer's promotion strategies are influenced by the commission factor and the promotional effort cost-sharing factor. When the latter factor is appropriate, it is more beneficial for the e-retailer to choose the cost-sharing strategy of promotional effort. In addition, the platform investing in promotional effort not only improves its profits, but also helps maximize the profits of the e-retailer. The study further finds that consumers acquire the maximum discounts when there is no promotional effort. As the promotional effort is invested, the discount declines and the demand rises instead.

## 1. Introduction

In recent years, e-commerce shopping festival has become the norm, as a key marketing promotion tool [1, 2]. In the early days, e-commerce platforms not only provided large subsidies to consumers, but also were very supportive and promotional for retailers, which in turn led to great success. This approach could attract e-retailers to participate, stimulate consumer demand, and enhance the overall activity of the platform. For instance, Alibaba Group's “Double Eleven” Shopping Carnival and JD.com's “618” Shopping Festival are the most popular and largest online shopping festivals in China.

The “Double Eleven” Shopping Festival, China's first online shopping festival, is a massive promotion conducted by Alibaba in 2009 on Singles' Day, the day of November 11th. The first time it was held, sales far exceeded expectations. Today, the single day has become the largest online shopping holiday in China. It is no coincidence that, in 2010, another e-commerce platform, JD.com, with its form features, launched the computer digital products promotion shopping festival, which is held on June 18th every year. As a result, it is known as the “618” Shopping Festival and has evolved into China's second largest comprehensive shopping event.  Table 1 shows the trading volume of the “Double Eleven” and the “618” shopping festivals in 2017–2021. The two shopping festivals have since been staged 13 times and have turned into an industry phenomenon, with additional e-commerce platforms joining the festivals.

However, as competition between e-commerce platforms has become increasingly fierce, the festivals have gradually deteriorated and bottlenecks have emerged. On the one hand, the frequency of shopping festivals is getting higher and higher, and the original low-price advantage is gradually disappearing. So consumers' attitudes towards shopping festivals are shifting from frenzy to rationality [3]. On the other hand, the participation of small and medium-sized e-retailers is gradually decreasing due to the little profit that they get from participating in promotional activities [4]. Therefore, how to break the bottlenecks of shopping festivals has become an urgent problem in the field of e-commerce. The platforms should actively play their platform advantages to avoid blindly creating festivals. At the same time, to achieve a “win-win” situation, they can incentivize and join hands with online retailers to provide discounts to consumers.

To effectively solve this problem, this paper intends to study how e-commerce platforms and e-retailers develop the optimal joint promotion strategy in terms of the cost-sharing factor of promotion effort. This work enriches and expands the research of promotion strategies in online shopping festivals and provides a reliable basis for both to make scientific decisions.

The remainder of this article is organized as follows. In Section 2, we present the literature review.  Section 3 sets forth the research hypotheses and introduces the models. In Section 4, we solve the models to obtain the optimal promotional discount, promotional effort, demand, and profit separately and also analyzed the effects of the factors on the variables. We perform a comparative analysis of these models in Section 5. Numerical simulations are in Section 6. Finally, Section 7 summarizes the findings of this paper and directions for further research.

## 2. Literature Review

Our work is closely related to two streams of literature: joint promotion and promotional effort.

The research on joint promotion first originated from Adler, who proposed that joint promotion between enterprises would lead to greater sales and be able to obtain more profits [5]. Many scholars have also pointed out that when two or more firms and brands conduct joint promotional activities, each member within the consortium can obtain greater promotional effects at lower cost [6–9]. However, a few scholars hold a different view, such as Vaidyanathan. He discovered that, in asymmetric brand copromotion relationships, brands in a weak position not only fail to gain but also allow profits to suffer [10]. Therefore, numerous scholars have further investigated various joint promotion strategies. For example, Geng and Mallik investigated the joint decisions of offering mail-in rebates (MIRs) in a single-manufacturer-single-retailer supply chain. It implied that a MIR makes a product look cheaper while the consumers pay more on average [11]. To help retailers accomplish the joint optimization of ordering and promotional strategies, Yang et al. analyzed two types of promotions, rebates and everyday low prices. They found that price sensitivity and regular undiscounted retail prices were among the most influential factors in choosing promotional strategies [12]. Darmawan et al. proposed a modeling framework for sales and operations planning that considers joint promotions and production planning for multiple products, to solve the resulting joint optimization problem [13]. To reveal the joint promotion mechanism of supply chain rebates under decentralized decision-making, Gu et al. investigated the retailer rebate and order joint decision and the supplier channel volume rebate decision [14]. Cao et al. studied a shared discount strategy in which retailers and consumers simultaneously divide discounts on products offered by manufacturers. In particular, they mainly discussed the impact of discount allocation ratios on optimal wholesale prices and retailers' ordering decisions [15]. To reveal the joint promotion mechanism of supply chain rebates under decentralized decision-making, Jiang et al. investigated the retailer rebate and order joint decision and the supplier channel volume rebate decision [16]. Jiang et al. studied the optimal joint promotion strategy for sellers and flattops under different game models and found that when sellers and flattops launch promotions independently at the same time, the overall supply chain promotion is the strongest and the overall profit of the supply chain is the highest [16]. All of the above studies only consider the promotion strategies between two subjects or products in the form of price, without considering that, in the actual sales process, there are other nonprice forms of promotions, which can also have a significant impact on consumer purchasing behavior, such as the promotional effort studied in this paper.

Regarding the study of promotional effort, John and Raj were the first to study the effect of promotional effort on sales. They argued that retailers could increase the level of promotional effort, which would benefit product sales. Since then, scholars have conducted extensive research [17]. Cárdenas-Barrón and Sana proposed an economic order quantity inventory model of multi-items in a two-layer supply chain where demand is sensitive to the promotional effort. They further suggested that, from a collaborative perspective, suppliers and retailers share the cost of promotional effort to increase profits for both parties [18]. Hosseini-Motlagh et al. analyzed the performance of a supply chain consisting of a monopolistic manufacturer and two competing retailers under a promotional effort credit-period dependent demand. They further researched and found that the centralized model had certain shortcomings, so they proposed a new collaborative model to not only increase the profitability of the whole supply chain but also ensure the participation of all members [19]. Huang and Bai analyzed the cooperative promotion between manufacturers and retailers in the presence of promotional effort. The study found that promotional effort makes manufacturers' profits increase and retailers' profits decrease [20]. To improve the firm's supply chain performance, Yu et al. explored the supply chain decision problem with demand-dependent promotional effort and variability. Through numerical analysis, they found that investing in promotional effort and reducing demand variability could increase the profitability of the centralized system [21]. Jia et al. established an information-sharing strategy for platforms under different promotion models. It mainly analyzed the impact of promotional effort cost coefficients between platforms and sellers on supply chain firms' decisions [22]. Gou et al. developed a two-channel supply chain coordination strategy model based on consistent pricing and promotional effort for the centralized and decentralized decision-making models, respectively. They further designed a network channel revenue-sharing contract with fixed compensation, which can realize the win-win benefits of each subject [23]. To summarize the above studies, we found that all of the literature examines the existence of promotional effort and lacks research on the extent of it.

In summary, existing literature has studied joint promotion and promotional effort more extensively but still has not been considered comprehensively. Therefore, this paper addresses the above issues by combining the two forms of promotions for a thorough study. And our setting is more relevant to the actual situation of e-commerce shopping festivals.

## 3. Model Description and Assumptions

In this study, an e-retailer sells products on an e-commerce platform. Among them, the platform is in a dominant position and receives a commission from the e-retailer through a revenue-sharing contract. During the e-commerce shopping festival, the platform invests a certain amount of promotional effort in publicity to stimulate consumers' purchase demand. At the same time, the platform and the retailer weigh their conditions to grant appropriate price discounts to consumers. The relevant assumptions are as follows.

**Hypothesis 1 .**

The market demand *D* for a product depends on price and promotional effort. Let the expression for market demand be *D*=*a* − *bp*+*ηe*.

In this formula, *a* is the potential market size. *b* is the price sensitivity coefficient of consumers. *η* is the sensitivity coefficient of consumers' promotional effort. Besides, *a*, *b*, and *η* are exogenous variables.

**Hypothesis 2 .**

According to the source, the discounts are divided into retailer's discounts (*α*) and platform's discounts (*β*). The amount of retailer's discounts is based on the selling price of the product, with a certain price discount factor for special promotions; for example, 200 minus 30 is equivalent to a 15% discount. Most of the platform's discounts are in the form of red packets, which are directly deducted from the product amount when placing an order. When a consumer uses the price discounts from the platform and the e-retailer, the amount he pays is *p* − *α* − *β*.

**Hypothesis 3 .**

Promotional effort is a general term for means and methods of promoting sales other than price. Reflected in e-commerce shopping festivals, it can be specifically expressed as advertising on major social media, holding shopping festival concerts, etc. The promotional effort coefficient of an e-commerce platform is indicated by *e*. We define the promotional effort cost as 1/2*ke*^2^ [23, 24], and *k* is the promotional effort cost factor. When the e-retailer chooses to bear part of the promotional effort cost, its cost-sharing ratio factor is *λ*. *k*, *λ* ∈ (0,1).

**Hypothesis 4 .**

An e-retailer needs to pay the commission to the platform in the proportion *f*, *f* ∈ (0,1), for each product sold.

**Hypothesis 5 .**

Each consumer buys at most one product, and all consumers are homogeneous and highly sensitive to promotional effort.

Table 2 shows the description of each symbol in this paper.

Based on the above assumptions, the e-commerce platform and the e-retailer contain a limited set of three strategies each in the online shopping festival. The platform can adopt the {No-Initiation *x*_1_, Initiation *x*_2_, Actively Initiation *x*_3_} strategy, where the “ Actively Initiation” strategy indicates that the platform invests promotional effort during the shopping festival, which can be concretely demonstrated by conducting a lot of advertising and holding promotional parties. The e-retailer can choose the {No-Participation *y*_1_, Participation *y*_2_, Actively Participation *y*_3_} strategy, where the “Actively Participation” strategy indicates that the retailer participates in the platform's promotional effort and shares the cost of the effort, allowing the retailer to engage in significant and consistent exposure on the platform's home page. Since the platform plays a dominant role, the six combinations of strategies in Table 3 can be formed. When the discount of the platform or the retailer is zero, it is the No-Initiation or No-Participation strategy. Therefore, the paper mainly analyzes three specific strategy models: the no-promotion-effort model (*x*_2_, *y*_2_), the platform-only promotion effort model (*x*_3_, *y*_2_), and the platform and e-retailer sharing promotion effort cost model (*x*_3_, *y*_3_).

## 4. Model Resolution

In this section, we solve the three promotional models described above and analyze the effect of each parameter on the decision variables.

### 4.1. Model 1: The No-Promotional-Effort Model (*x*_2_, *y*_2_)

This subsection analyzes the case where the e-commerce platform and the retailer only offer price discounts. At this point, the functions of demand and profits are expressed as

(1) $$\begin{array}{c} D_{1}=a-b\left(p-\alpha_{1}-\beta_{1}\right)\text{,} \end{array}$$

(2) $$\begin{array}{c} \text{,} \end{array}$$

(3) $$\begin{array}{c} \text{.} \end{array}$$

The optimal decision of this model is solved by using Backward Induction.

First, we can obtain the first-order and second-order partial derivatives of *π*_1*R*_ with respect to *α*_1_ from equation (2).

(4) $$\begin{array}{c} \begin{array}{c} \frac{\partial \pi_{1R}}{\partial \alpha }=-\left(1-f\right)\left[a-b\left(p-\alpha_{1}-\beta_{1}\right)\right]+\left[\left(1-f\right)\left(p-\alpha_{1}\right)-c\right]b,\\ \begin{array}{c} \frac{\partial^{2}\pi_{1R}}{\partial \alpha_{1}^{2}}=-2\left(1-f\right)b<0 \end{array}. \end{array} \end{array}$$

Since *∂*^2^*π*_1*R*_/*∂α*_1_^2^ < 0, there is a unique optimal solution *α*_1_^*∗*^ that maximizes *π*_1*P*_. Let *∂π*_1*R*_/*∂α*=0 to find the optimal price discount for the e-tailer *α*_1(*β*_1_)_^*∗*^.

(5) $$\begin{array}{c} \begin{array}{c} {\alpha_{1}}_{\left(\beta_{1}\right)}^{*}=p-\frac{\left(1-f\right)\left(a+b\beta_{1}\right)+cb}{2b\left(1-f\right)} \end{array}\text{.} \end{array}$$

Second, substituting equation (5) into equation (3), we obtain the profit of the e-commerce platform.

(6) $$\begin{array}{c} \begin{array}{c} \pi_{1P}=\left[\frac{af\left(1-f\right)-\left(1-f\right)\left(2-f\right)b\beta_{1}+bcf}{2b\left(1-f\right)}\right]\left[\frac{\left(1-f\right)\left(a+b\beta_{1}\right)-bc}{2\left(1-f\right)}\right] \end{array}\text{.} \end{array}$$

At this point, the profit function of the platform is a quadratic function of the discounts *β*_1_. Then find its first-order and second-order derivatives with respect to *β*_1_.

(7) $$\begin{array}{c} \begin{array}{c} \frac{\partial \pi_{1P}}{\partial \beta_{1}}=\frac{bc-a{\left(1-f\right)}^{2}-\left(1-f\right)\left(2-f\right)b\beta }{2\left(1-f\right)},\\ \frac{\partial^{2}\pi_{1P}}{\partial \beta_{1}^{2}\;}=-\frac{\left(2-f\right)b}{2}<0. \end{array} \end{array}$$

Since *∂*^2^*π*_1*P*_/*∂β*_1_^2^ < 0, there is a unique optimal solution *β*_1_^*∗*^ that maximizes *π*_1*P*_. The optimal price discount for the platform can be found by letting *∂π*_1*P*_/*∂β*_1_=0.

Finally, substituting the result into equations (1)–(3), (5), the optimal solution for the case is obtained as follows:

(8) $$\begin{array}{c} \alpha_{1}^{*}=p-\frac{a\left(1-f\right)+bc\left(3-f\right)}{2b\left(1-f\right)\left(2-f\right)}\text{,}\\ \beta_{1}^{*}=\frac{bc-a{\left(1-f\right)}^{2}}{b\left(1-f\right)\left(2-f\right)}\text{,}\\ D_{1}^{*}=\frac{a-bc}{2\left(2-f\right)}\text{,}\\ \pi_{1R}^{*}=\frac{\left(1-f\right){\left(a-bc\right)}^{2}}{4b{\left(2-f\right)}^{2}}\text{,}\\ \pi_{1P}^{*}=\frac{{\left(a-bc\right)}^{2}}{4b\left(2-f\right)}\text{.} \end{array}$$

By analyzing the optimal solution of the model 1, we can obtain several propositions.

**Proposition 1 .**

When the commission coefficient $f>1-\sqrt{bc/a}$, the platform will choose to take the lead in launching the shopping festival. Whether the retailer chooses to follow and participate in the joint promotion depends on the price and commission. When the cost, price, and commission satisfy the condition *p* > *a*(1 − *f*)+*bc*(3 − *f*)/2*b*(1 − *f*)(2 − *f*), the retailer will choose to participate in the shopping festival.

**Proof**

The equilibrium solution for the price discounts in model 1 is known to be *α*_1_^*∗*^=*p* − *a*(1 − *f*)+*bc*(3 − *f*)/2*b*(1 − *f*)(2 − *f*), *β*_1_^*∗*^=*bc* − *a*(1 − *f*)^2^/*b*(1 − *f*)(2 − *f*), and *α*, *β* > 0. Therefore, solving *α*_1_^*∗*^ > 0, *β*_1_^*∗*^ > 0.  Proposition 1 is proved.

Proposition 1 states that an e-commerce platform chooses to launch an online shopping festival only when the commission factor is above a certain threshold. The reason for this is that the platform derives its revenue mainly from the commissions of retailers. When the revenue is large, the platform will choose to spend a certain commission and launch a festival to stimulate consumption, which in turn will increase the activity of the platform and gain more profit. Whether or not the retailer participates in the festival depends on the selling price and commission factor of the products. When the price is higher or the commission factor is lower, retailer will participate in joint promotions with the platform, but not vice versa.

**Proposition 2 .**

In the case of e-commerce platforms and online retailer offering only price discounts, *α*_1_^*∗*^ and *π*_1*R*_^*∗*^ are negatively correlated with *f*; *β*_1_^*∗*^ and *π*_1*P*_^*∗*^ are positively correlated with *f*; *α*_1_^*∗*^ is negatively correlated with *β*_1_^*∗*^.

**Proof**

Find, respectively, the first-order derivative of *α*_1__(*β*_1_)_^*∗*^, *α*_1_^*∗*^, *β*_1_^*∗*^, *π*_1*R*_^*∗*^, *π*_1*P*_^*∗*^ with respect to *f*. *∂α*_1_^*∗*^/*∂f*=−2*ab*(1 − *f*)^2^ − 2*b*^2^*c*(*f*^2^ − 6*f*+7). Since *f* ∈ [0,1], it is known that *f*^2^ − 6*f*+7 takes values in the range [2, 7], so *∂α*_1_^*∗*^/*∂f* < 0; *∂π*_1*R*_^*∗*^/*∂f*=−(*a* − *bc*)^2^/4*b*(2 − *f*)^3^ < 0; *∂β*_1_^*∗*^/*∂f*=*ab*(1 − *f*)^2^+*b*^2^*c*(3 − 2*f*) > 0; =*∂π*_1*P*_^*∗*^/*∂f*4*b*(*a* − *bc*)^2^/4*b*(2 − *f*) > 0; *∂α*_1__(*β*_1_)_^*∗*^/*∂β*_1_=−1/2(1 − *f*) < 0.

Proposition 2 shows that when an e-commerce platform initiates a shopping festival and an e-retailer participates, the price discounts and profits of both are affected by the commission factor. For the platform, the discounts *β*_1_^*∗*^ and profit *π*_1*P*_^*∗*^ are positively proportional to the commission factor *f*. However, for the e-retailer, the discounts *α*_1_^*∗*^ and profit *π*_1*R*_^*∗*^ are inversely proportional to the commission factor *f*. In addition, the amount of discounts for the retailer *α*_1_^*∗*^ is inversely proportional to the amount of discounts for the platform *β*_1_^*∗*^.

For an online retailer, the larger the commission factor is, the larger the commission it needs to pay to the platform and the less net profit it receives. If it still offers larger discounts, it will not be able to make a profit. However, for e-commerce platforms, the more commissions they charged, the bigger the profit, and the more discount subsidies they are willing to give to consumers. Furthermore, since most consumers do not purposely distinguish the source of promotional discounts, they only focus on the final out-of-pocket amount. Therefore, when more discounts are observed to be invested by the platform, the e-retailer will appropriately reduce price discounts and lower this part of the loss to gain more profit.

### 4.2. Model 2: The Platform-Only Promotion Effort Model (*x*_3_, *y*_2_)

This subsection analyzes a combination strategy in which the e-commerce platform actively initiates and the e-retailer participates. At this point, e-commerce platforms need to spend promotional effort cost. The demand and the profits are as follows:

(9) $$\begin{array}{c} \begin{array}{c} D_{2}=a-b\left(p-\alpha_{2}-\beta_{2}\right)+\eta e_{2} \end{array}\text{,} \end{array}$$

(10) $$\begin{array}{c} \begin{array}{c} \pi_{2R}=\left[\left(1-f\right)\left(p-\alpha_{2}\right)-c\right]\left[a-b\left(p-\alpha_{2}-\beta_{2}\right)+\eta e_{2}\right] \end{array}\text{,} \end{array}$$

(11) $$\begin{array}{c} \begin{array}{c} \pi_{2P}=\left[f\left(p-\alpha_{2}\right)-\beta_{2}\right]\left[a-b\left(p-\alpha_{2}-\beta_{2}\right)+\eta e_{2}\right]-\frac{1}{2}ke_{2}^{2} \end{array}\text{.} \end{array}$$

As in subsection 4.1, the optimal decision for both is analyzed using Backward Induction. From equation (10), we can obtain the first-order and second-order partial derivatives of *π*_2*R*_ with respect to *α*_2_.

Since *∂*^2^*π*_2*R*_/*∂α*_2_^2^=−2(1 − *f*)*b* < 0, there is a unique optimal solution *α*_2_^*∗*^ that maximizes *π*_2*R*_. Let *∂π*_2*R*_/*∂α*_2_=0 to find the optimal price discount for the e-tailer.

(12) $$\begin{array}{c} \begin{array}{c} {\alpha_{2}}_{\left(\beta_{2},e_{2}\right)}^{*}=p-\frac{\left(1-f\right)\left(a+b\beta_{2}+\eta e_{2}\right)+bc}{2b\left(1-f\right)} \end{array}\text{.} \end{array}$$

Then, substituting equation (12) into equation (11), we obtain the profit function of the e-commerce platform as a quadratic function of its price discounts and promotional effort.

(13) $$\begin{array}{c} \begin{array}{c} \pi_{2P}=\frac{1}{2}\left[f\left(\frac{\left(1-f\right)\left(a+b\beta_{2}+\eta e_{2}\right)+bc}{2b\left(1-f\right)}\right)-\beta_{2}\right]\left[a-\frac{bc}{1-f}+b\beta_{2}+\eta e_{2}\right]-\frac{1}{2}ke_{2}^{2} \end{array}\text{.} \end{array}$$

The first-order partial derivatives and second-order partial derivatives of *π*_2*P*_ with respect to *β*_2_ and *e*_2_ are as follows:

(14) $$\begin{array}{c} \begin{array}{c} \frac{\partial \pi_{2P}}{\partial \beta_{2}}=\frac{bc-{\left(1-f\right)}^{2}\left(a+\eta e\right)-\left(1-f\right)\left(2-f\right)b\beta }{2\left(1-f\right)} \end{array}\text{,} \end{array}$$

(15) $$\begin{array}{c} \begin{array}{c} \frac{\partial \pi_{2P}}{\partial e_{2}}=\frac{\left(a+\eta e\right)f\eta -\left(1-f\right)b\eta \beta -2bke}{2b} \end{array}\text{.} \end{array}$$

Its Hesse matrix is given by

(16) $$\begin{array}{c} \left|\begin{array}{cc} \frac{\partial^{2}\pi_{2P}}{\partial \beta_{2}^{2}} & \frac{\partial^{2}\pi_{2P}}{\partial \beta_{2}\partial e_{2}}\\ \frac{\partial^{2}\pi_{2P}}{\partial e_{2}\partial \beta_{2}} & \frac{\partial^{2}\pi_{2P}}{\partial e_{2}^{2}} \end{array}\right|=\frac{1}{2}bk\left(2-f\right)-\frac{1}{4}\eta^{2} \end{array}$$

It is known that *π*_2*P*_ is a concave function of *β*_2_ and *e*_2_ when the condition 2*bk*(2 − *f*) − *η*^2^ > 0 is satisfied. The optimal price discount *β*_2_^*∗*^ and optimal promotion effort *e*_2_^*∗*^ can be obtained by solving equations (14) and (15). Substituting the results into equations (9)–(12), we obtain the following optimal solution:

(17) $$\begin{array}{c} \alpha_{2}^{*}=p-\frac{ak\left(1-f\right)+bck\left(3-f\right)-c\eta^{2}}{\left(1-f\right)\left[2bk\left(2-f\right)-\eta^{2}\right]}\text{,}\\ \beta_{2}^{*}=\frac{2bck-2ak{\left(1-f\right)}^{2}-cf\eta^{2}}{\left(1-f\right)\left[2bk\left(2-f\right)-\eta^{2}\right]}\text{,}\\ e_{2}^{*}=\frac{\eta \left(a-bc\right)}{2bk\left(2-f\right)-\eta^{2}}\text{,}\\ D_{2}^{*}=\frac{\left(a-bc\right)bk}{2\left(2-f\right)bk-\eta^{2}}\text{,}\\ \pi_{2R}^{*}=\frac{{\left(a-bc\right)}^{2}\left(1-f\right)bk^{2}}{{\left[2bk\left(2-f\right)-\eta^{2}\right]}^{2}}\text{,}\\ \pi_{2P}^{*}=\frac{{\left(a-bc\right)}^{2}k}{2\left[2bk\left(2-f\right)-\eta^{2}\right]}\text{.} \end{array}$$

By analyzing the optimal solution of the model 2, we can get Propositions 3 and 4.

**Proposition 3 .**

When the commission coefficient satisfies *f* < 2 − *η*/2*bk* and 2*ak*(1 − *f*)^2^+*cfη*^2^ < 2*bck*, the platform will actively launch a shopping festival and invest in the promotional effort. The retailer will participate in the festival when the price of the product and the commission factor satisfy the condition *p* > *ak*(1 − *f*)+*bck*(3 − *f*) − *cη*^2^/(1 − *f*)[2*bk*(2 − *f*) − *η*^2^]. The proof is the same as Proposition 1.

**Proposition 4 .**

In model 2, *β*_2_^*∗*^, *e*_2_^*∗*^, *π*_2*P*_^*∗*^ are positively correlated with *f*; *α*_2_^*∗*^ is negatively correlated with *f*; when *f* ≤ *η*^2^/2*bk*, *π*_2*R*_^*∗*^ is positively correlated with *f*, and vice versa. The proof is the same as Proposition 2.

Proposition 3 analyzes the conditions for the portfolio strategy {Actively Initiation *x*_3_, Participation *y*_2_} and Proposition 4 analyzes the effect of the commission coefficient on each variable. The similarities with the results of model 1 are not repeated here. The difference between the findings of model 2 is the effect of the commission factor on the profitability of the retailer. When the commission factor is less than a certain threshold, the retailer's profit will first increase, but when it exceeds that value, the profit will decrease. This is because the platform spends part of its commissions on promotional effort for e-commerce shopping festivals, which stimulates consumer demand and increases optimal demand, thus increasing the retailer's profits. However, when the commission is too large, the increased profit is not enough to offset the extra commission paid, so the profit tends to decrease.

### 4.3. Model 3: The Platform and e-Retailer Sharing Promotion Effort Cost Model (*x*_3_, *y*_3_)

This subsection analyzes the combination strategy in which the e-commerce platform actively initiates and the e-retailer actively participates. At this point, the retailer bears some of the cost of the platform's promotional effort. The demand and the profits are as follows:

(18) $$\begin{array}{c} \begin{array}{c} \pi_{3R}=\left[\left(1-f\right)\left(p-\alpha_{3}\right)-c\right]\left[a-b\left(p-\alpha_{3}-\beta_{3}\right)+\eta e_{3}\right]-\frac{1}{2}\lambda ke_{3}^{2}, \end{array} \end{array}$$

(19) $$\begin{array}{c} \text{.} \end{array}$$

Model 3 is solved in the same procedure as model 2.

*∂* ^2^ *π*_3*R*_/*∂α*_3_^2^=−2(1 − *f*)*b* < 0, and thus there exists a unique optimal solution *α*_3_^*∗*^ such that *π*_3*R*_ is maximized. Therefore, let *∂π*_3*R*_/*∂α*_3_=0; the e-tailer's optimal price discount *α*_3__(*β*_3_, *e*_3_)_^*∗*^ is obtained.

(20) $$\begin{array}{c} \begin{array}{c} {\alpha_{3}}_{\left(\beta_{3},e_{3}\right)}^{*}=p-\frac{\left(1-f\right)\left(a+b\beta_{3}+\eta e_{3}\right)+bc}{2b\left(1-f\right)} \end{array}\text{.} \end{array}$$

Substituting equation (19) into equation (18), we obtain the expression of *π*_3*P*_ with respect to *β*_3_, *e*_3_. The Hesse matrix is given by

(21) $$\begin{array}{c} \begin{array}{c} \left|\begin{array}{cc} \frac{\partial^{2}\pi_{3P}}{\partial \beta_{3}^{2}} & \frac{\partial^{2}\pi_{3P}}{\partial \beta_{3}\partial e_{3}}\\ \frac{\partial^{2}\pi_{3P}}{\partial e_{3}\partial \beta_{3}} & \frac{\partial^{2}\pi_{3P}}{\partial e_{3}^{2}} \end{array}\right|=\frac{1}{2}\left(2-f\right)\left(1-\lambda \right)bk-\frac{1}{4}\eta^{2} \end{array}\text{.} \end{array}$$

It is known that when 2(2 − *f*)(1 − *λ*)*bk* − *η*^2^ > 0, there is an optimal solution. The e-commerce platform's optimal price discount and the optimal promotion effort can be obtained by solving *∂π*_3*P*_/*∂β*_3_=0 and *∂π*_3*P*_/*∂e*_3_=0. In turn, we obtain the optimal price discount of the e-tailer, the optimal demand, the optimal profit of the e-tailer, and the platform.

(22) $$\begin{array}{c} \alpha_{3}^{*}=p-\frac{ak\left(1-f\right)\left(1-\lambda \right)+bck\left(3-f\right)\left(1-\lambda \right)-c\eta^{2}}{\left(1-f\right)\left[2bk\left(2-f\right)\left(1-\lambda \right)-\eta^{2}\right]}\text{,}\\ \beta_{3}^{*}=\frac{2bck\left(1-\lambda \right)-2ak{\left(1-f\right)}^{2}\left(1-\lambda \right)-cf\eta^{2}}{\left(1-f\right)\left[2bk\left(2-f\right)\left(1-\lambda \right)-\eta^{2}\right]}\text{,}\\ e_{3}^{*}=\frac{\eta \left(a-bc\right)}{2bk\left(2-f\right)\left(1-\lambda \right)-\eta^{2}}\text{,}\\ D_{3}^{*}=\frac{\left(a-bc\right)\left(1-\lambda \right)bk}{2\left(2-f\right)\left(1-\lambda \right)bk-\eta^{2}}\text{,}\\ \pi_{3R}^{*}=\frac{{k\left(a-bc\right)}^{2}\left[2\left(1-f\right){\left(1-\lambda \right)}^{2}bk-\lambda \eta^{2}\right]}{2{\left[2bk\left(2-f\right)\left(1-\lambda \right)-\eta^{2}\right]}^{2}}\text{,}\\ \pi_{3P}^{*}=\frac{\left(1-\lambda \right){\left(a-bc\right)}^{2}k}{2\left[2bk\left(2-f\right)\left(1-\lambda \right)-\eta^{2}\right]}\text{.} \end{array}$$

By analyzing the optimal solution of the model 1, we can get the following propositions.

**Proposition 5 .**

When the commission factor and promotion effort cost-sharing factor meet the two conditions *f* < 2 − *η*/2*bk*(1 − *λ*) and *λ* < 1 − *cfη*^2^/2*bck* − 2*ak*(1 − *f*)^2^, the platform will actively launch a shopping festival. When price, commission factor, and cost-sharing factor satisfy the inequality *p* > *ak*(1 − *f*)(1 − *λ*)+*bck*(3 − *f*)(1 − *λ*) − *cη*^2^/(1 − *f*)[2*bk*(2 − *f*)(1 − *λ*) − *η*^2^], the retailer will actively participate in the festival. The proof is the same as Proposition 1.

**Proposition 6 .**

In model 3, the effect of the commission coefficient on each variable: *β*_3_^*∗*^, *e*_3_^*∗*^, *π*_3*P*_^*∗*^ are positively correlated with *f*. *α*_3_^*∗*^ is negatively correlated with *f*. When *f* ≤ 4*bkλ*(1 − *λ*)^2^+(1 − 4*λ*+*λ*^2^)*η*^2^/2*bk*(1+*λ*)(1 − *λ*)^2^, *π*_3*R*_^*∗*^ is positively correlated with *f*, and vice versa. Then the effect of sharing coefficients on each variable: *e*_3_^*∗*^ and *π*_3*P*_^*∗*^ are positively correlated with *λ*. *α*_3_^*∗*^ and *β*_3_^*∗*^ are negatively correlated with *λ*. When *λ* > 2(1 − *f*)(2 − *f*)*b*^2^*k* − *bkη*^2^+1/4*η*^2^/*b*^2^*k*^2^+(2 − *f*)*bkη*^2^, *π*_3*P*_^*∗*^ is positively correlated with *λ*, and vice versa. The proof is the same as Proposition 2.

Propositions 5 and 6 show that the effect of the commission factor on the variables is consistent with Propositions 3 and 4 in model 2. Concerning the effect of the cost-sharing coefficient on each variable, the joint promotion of both will decrease when the cost-sharing coefficient is larger. However, promotional effort and profits will increase. For the retailer, the profit tends to increase and then decrease.

When the retailer shares the cost of promotional effort on the platform, the platform will increase its promotional effort. Both of them will reduce the discounts offered to consumers. This suggests that, for consumers, promotional effort will indirectly reduce the substantial merchandise discounts. Moreover, when the cost-sharing coefficient is in a desirable range, e-retailer's sharing of the platform's promotional effort can increase their profits, but profits will decline when the cost-sharing coefficient is larger. This explains why so many brands choose to sponsor, implant, and title the shopping festival concerts on major e-commerce platforms.

## 5. Promotion Model Comparison

This section analyzes the relationship between joint promotion, promotional effort, demand, and profit under three types of promotional models.

**Proposition 7 .**

In the three-type promotion model, when the retailer and the platform jointly promote, the optimal discount for the e-tailer: *α*_1_^*∗*^ > *α*_2_^*∗*^ > *α*_3_^*∗*^, the optimal discount for the platform: *β*_1_^*∗*^ > *β*_2_^*∗*^ > *β*_3_^*∗*^, and the optimal promotion effort: *e*_2_^*∗*^ < *e*_3_^*∗*^. The proof process is simple and will not be repeated here.

Proposition 7 illustrates that, for both e-retailer and e-commerce platform, sharing promotional effort will cause the platform to invest more in the promotional effort, but both will reduce the discounts of joint promotion. Therefore, for consumers, when the e-commerce shopping festival has only joint promotions without any promotional effort, consumers get the most discounts from it. This is also as expressed in Proposition 6.

**Proposition 8 .**

In the three promotion models, the optimal demand: *D*_1_^*∗*^ < *D*_2_^*∗*^ < *D*_3_^*∗*^.

Combined with Proposition 7, Proposition 8 illustrates that, in online shopping festival, although promotional effort reduces discounts, it will still greatly stimulate consumer demand. Consumers enjoy the lively atmosphere of the shopping festival and are not explicitly aware of the cut in discounts. At the same time, for the e-retailer, sharing the cost of promotional effort on the platform can effectively increase sales, achieve clearance, and prepare for new products to be stocked.

**Proposition 9 .**

Comparing the profits of e-tailers and e-commerce platforms for three promotional models. The optimal profit for the e-tailer: when *λ* < 2 − [2*bk*(2 − *f*) − *η*^2^]^2^/(1 − *f*)*bkη*^2^, *π*_3*R*_^*∗*^ > *π*_2*R*_^*∗*^ > *π*_1*R*_^*∗*^; when *λ* > 2 − [2*bk*(2 − *f*) − *η*^2^]^2^/(1 − *f*)*bkη*^2^, *π*_2*R*_^*∗*^ > *π*_3*R*_^*∗*^ > *π*_1*R*_^*∗*^. The optimal profit for the e-commerce platform: *π*_3*P*_^*∗*^ > *π*_2*P*_^*∗*^ > *π*_1*P*_^*∗*^.

Proposition 9 shows that when the commission factor and the cost-sharing factor of promotional effort satisfy a certain condition, the participation of the retailer in the promotional effort will lead to an increase in the profits of both. However, if the commission factor or the cost-sharing factor is too large, the retailer's profit will decrease. For the platform, the profit gained will increase although it needs to spend extra costs to initiate promotional effort. Therefore, when the two factors are appropriate, the e-commerce platform and the e-retailer choose to share the cost of promotional effort in favor of higher profits.

## 6. Numerical Simulation

To further verify the validity of the above findings and understand the impact of each parameter on the promotional strategies of the e-retailer and the e-commerce platform, this section takes the “Double Eleven” Shopping Festival as an example and then uses MATLAB to simulate three promotional models.

In the “Double Eleven” shopping festival, consumers can participate not only in the platform's subsidy activities, but also in the retailer's promotional activities. Both can directly offset the product amount. For example, if the daily pricing of a pair of sneakers is 499 RMB, the consumer gets the platform bonus of 15 RMB, and the discount of the retailer is 90 RMB, and then the actual final payment amount of the sneakers purchased by the consumer in the “Double Eleven” shopping festival is 394 RMB.

In conjunction with the above example, and in order not to lose generality, we assign values to the parameters: *a*=200, *b*=4, *η*=3, *p*=50, *c*=25, *k*=5. Keeping other parameters constant, take *λ*=0.3, *f* ∈ (0, 1) to analyze the effect of the commission coefficient on each variable. Take *f*=0.35, *λ* ∈ (0, 0.86) to analyze the effect of the promotional effort sharing coefficients on each variable, where the range of values satisfies the conditions for the existence of optimal solutions in models 2 and 3.

### 6.1. Optimal Joint Promotion and Promotional Effort

This subsection analyzes, respectively, the impact of the commission factor and the promotional effort cost-sharing factor on the strength of promotional discounts and promotional effort.

**Figure 1:**
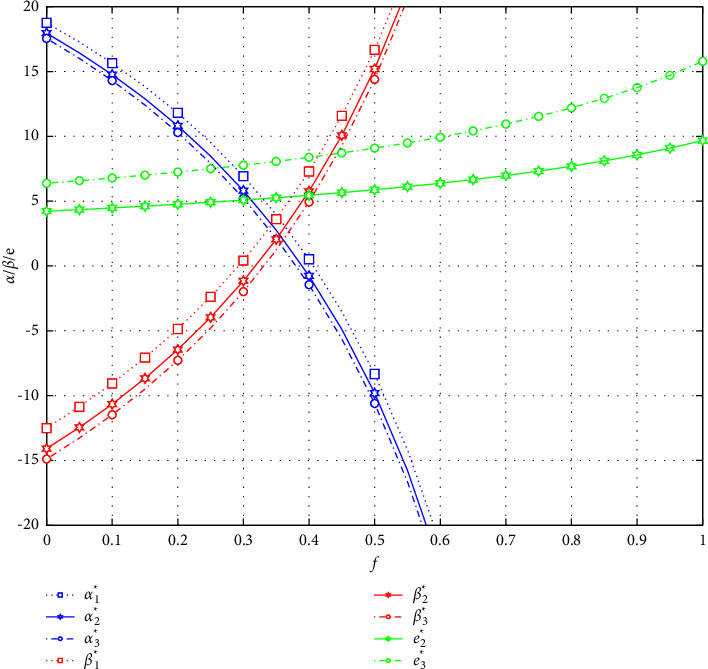
Impact of the commission factor on discounts and promotional effort.

As shown in Figure 1, for the platform, when the commission factor *f* < 0.3, they will not launch an e-commerce shopping festival. For the e-retailer, when the commission factor *f* > 0.4, they will not participate in the festival. Regardless of the promotional model, the impact of the commission factor on the discounts and the promotional effort is the same. As the commission factor increases, the retailer's promotional discounts will decrease, while the platform's promotional discounts and promotional effort will increase. Furthermore, observing the green curves, we could find that when the e-retailer chooses to share the promotional effort, the platform will invest more promotional effort.

**Figure 2:**
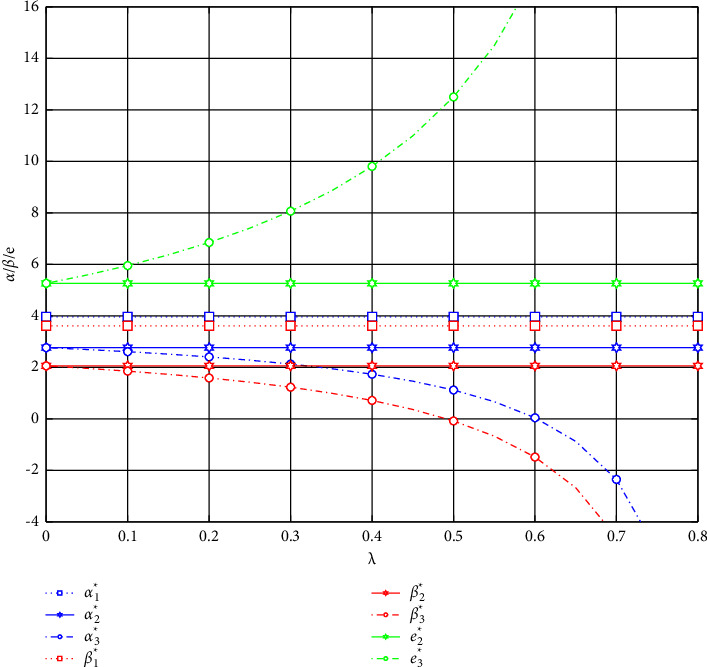
Impact of the cost-sharing factor on discounts and promotional effort.

As shown in Figure 2, for the e-tailer, when the promotional effort cost-sharing factor *λ* > 0.6, it will no longer offer promotional discounts to consumers. For the platform, when the e-tailer shares more than 50% of the promotional effort, it will no longer offer promotional discounts to consumers. As the cost-sharing factor increases, the promotional effort of the platform will increase, but the joint promotional discounts will decrease.

### 6.2. Optimal Profits

This subsection analyzes the impact of the commission factor and promotional effort cost-sharing factor on the optimal profitability of retailer and platform.

**Figure 3:**
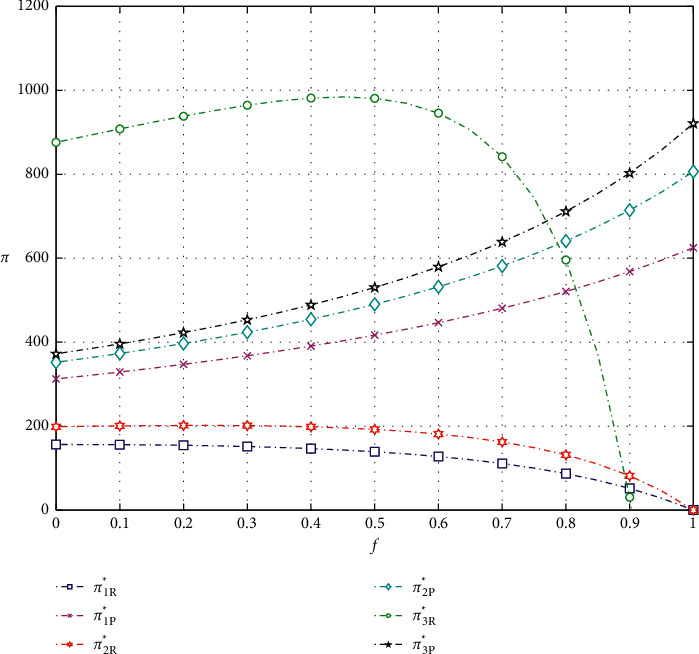
Impact of the commission factor on optimal profits.

**Figure 4:**
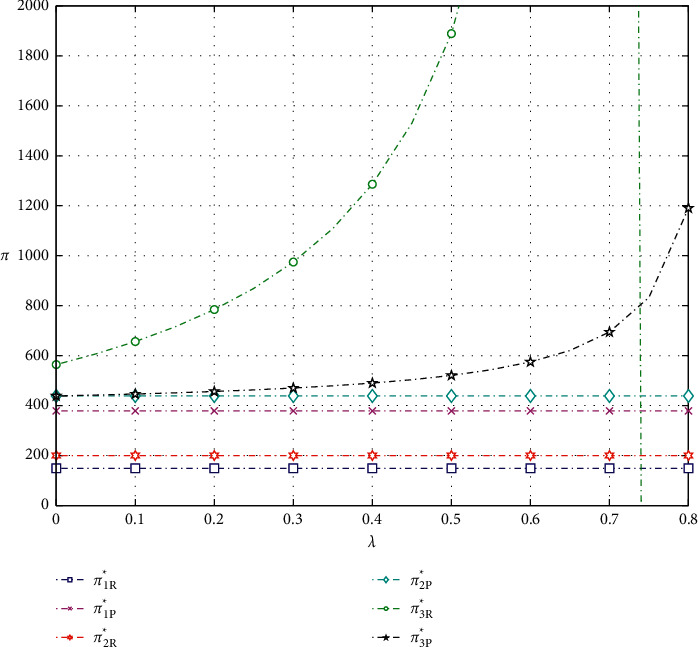
Impact of the cost-sharing factor on optimal profits.

As shown in Figures 3 and 4, the profit of the e-commerce platform increases as the commission factor and the promotional effort sharing factor increase.

**Figure 5:**
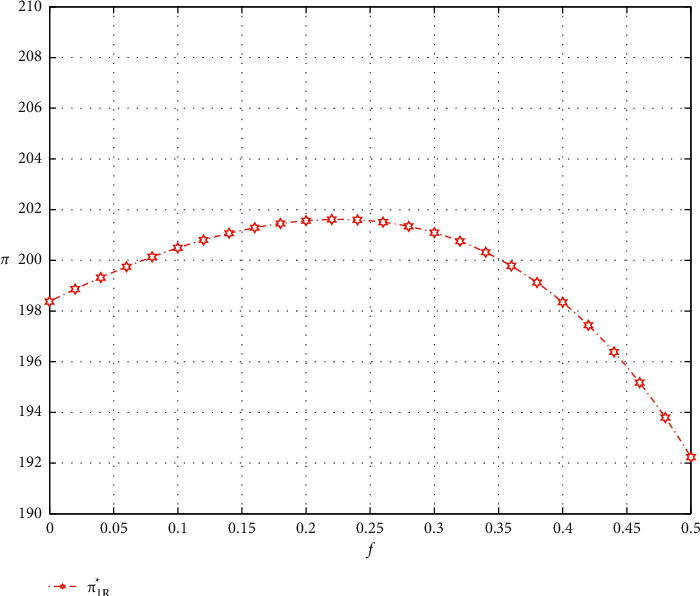
Impact of the commission factor on the e-retailer' optimal profit (partial).

As for e-retailer, the optimal profit in model 1 decreases as the commission factor increases. But, in models 2 and 3, the optimal profit tends to increase and then decrease. As shown in Figures 3 and 5, when the commission factor *f* approaches 0.225 in model 2 and approaches 0.45 in model 3, the profit of the e-tailer reaches the maximum. It is clear to observe that the optimal profit of the e-retailer choosing promotion model 3 is much higher than that of models 1 and 2 when the commission coefficient is not very large. Finally, we can also find that the profit of the e-retailer increases first with the increase of the promotional effort sharing coefficient, but then the profit plummets, as shown in Figure 4.

## 7. Conclusion

Considering the cost-sharing problem of platform-driven promotion effort, this study investigates the optimal joint promotion strategies of one e-commerce platform and one e-retailer. By building three different joint promotion models, we analyze the effects of the commission coefficient and promotion effort cost-sharing coefficient on the decisions of the platform and the e-retailer. This paper provides innovative ideas and a scientific basis for the promotion models of online shopping festivals. The results of the study are as follows.

1. Only when the commission factor and the selling price of goods are in a certain range, the platform will carry out the online shopping festivals, and the e-retailer will choose the strategy of following. Therefore, platforms should rationalize these factors to the profitability of the system without losing their own profits. Retailers should participate in the festivals cautiously after a reasonable analysis of the indicators.
2. The decisions of the platform and the e-retailer are influenced by the commission coefficient and the cost-sharing coefficient of promotional effort. In the three promotion models, the impact of the commission coefficient on discounts, promotional effort, demand, and the profitability of the platform is consistent. In contrast, the profits of the e-retailer show different trends with the commission coefficient.
3. For the e-commerce platform, choosing an aggressive initiation strategy, which means investing in promotional effort, not only effectively increases its profits, but also helps maximize the profits of e-retailers to achieve mutual benefits. This suggests that platforms should actively take a leading role in actively propagating the shopping festival for the mutual benefit of platforms and retailers.
4. For the e-tailer, when the commission factor is too large or the profit per unit of product is small, it is better to maintain the original profit by not participating in the shopping festival. When the commission factor is moderate, choosing to participate will increase the profit slightly. When the cost-sharing coefficient of promotional effort is appropriate or when the purpose is to clear inventory, it is most advantageous for it to share the promotional effort strategy of the platform. This strategy can not only effectively reduce the original inventory, but also enhance brand awareness and gain great profits.
5. For consumers, they receive maximum promotional discounts when there is no promotional effort. The more promotional effort platform put in, the less discount consumers get. Consumers should be sensible in distinguishing the extent of promotional discounts and not be lured into spending by overwhelming advertisements.

However, this study has some shortcomings that need to be addressed. First, this paper addresses the situation where a single retailer is selling on an e-commerce platform. But the market is in a competitive environment. In the future, we can study the situation of multiple e-tailers or multiple e-commerce platforms. Second, it is assumed in the paper that consumers are homogeneous. All consumers are highly sensitive to promotional effort. But in reality, consumers are a heterogeneous group. Next, we can examine the situation in which consumers are heterogeneous.

## Figures and Tables

**Table 1:** The trading volume of the “Double Eleven” and the “618” shopping festivals in 2017–2021. (Unit: 100 million yuan).

Year	2017	2018	2019	2020	2021
Turnover of “Double Eleven”	1682	2135	2684	4982	5403
Turnover of “618”	1199	1592	2015	2692	3438

Source: Alibaba and JD official website.

**Table 2:** Notations description.

Notations	Definitions
*R*	The e-retailer
*P*	The e-commerce platform
*c*	Cost per unit of product
*p*	Price per unit of product
*D*	Consumer demand
*α*	Amount of discounts for the e-retailer
*β*	Amount of discounts for the e-commerce platform
*e*	Promotion effort factor of the e-commerce platform
*f*	Commission factor
*λ*	Promotional effort cost-sharing ratio factor
*π*_*iR*_	The profit of the e-retailer. *i* denotes the number of models
*π*_*iP*_	The profit of the e-commerce platform

**Table 3:** The strategy portfolio matrix for the e-commerce platform and the e-tailer.

		The retailer		
		*y*_1_	*y*_2_	*y*_3_
The platform	*x*_1_	(*x*_1_, *y*_1_)	/	/
	*x*_2_	(*x*_2_, *y*_1_)	(*x*_2_, *y*_2_)	/
	*x*_3_	(*x*_3_, *y*_1_)	(*x*_3_, *y*_2_)	(*x*_3_, *y*_3_)

## Data Availability

The data used to support the findings of this study can be obtained from the corresponding author upon request.
